# The roll-to-roll revolution to tackle the industrial leap for perovskite solar cells

**DOI:** 10.1038/s41467-024-48518-4

**Published:** 2024-05-11

**Authors:** Ershad Parvazian, Trystan Watson

**Affiliations:** SPECIFIC IKC, Faculty of Science and Engineering, Swansea University, Fabian way, Swansea, SA1 8EN UK

**Keywords:** Solar cells, Devices for energy harvesting

## Abstract

Roll-to-Roll (R2R) coating is a technology that potentially enhances throughput, reduces costs, and accommodates flexible substrates for fabricating various types of solar cells and modules. Here, authors discuss the R2R revolution to tackle the industrial leap for perovskite photovoltaic devices.

## Teaser

Can Roll-to-Roll technology pave the way for perovskite devices to transition from lab-scale to industrial applications? It is a technique that has the potential to enhance throughput, reduce costs, and accommodate flexible substrates. In a Roll-to-Roll system, as long as your input materials are continuously topped up, manufacture should continue indefinitely. In its simplest form it offers the premise of Ink-IN / Solar module-OUT. Through this comment, we emphasize the critical need for ongoing innovation to fully harness Roll-to-Roll technology’s capabilities, making perovskite solar energy a viable, sustainable option on a global scale.

## R2R coating: revolutionizing perovskite PV technology

Roll-to-Roll (R2R) coating is distinguished by its efficient process in which inks are continuously deposited onto flexible substrates, which are injected through the system in a constant, unbroken stream, eliminating the need for manual substrate replacement and thereby differentiating it from discrete, sheet-to-sheet (S2S) methods^1^. This technique transforms the manufacturing landscape of solar cell production, including perovskite photovoltaic (PV) technologies, by significantly lowering costs, achieved through a continuous, efficient process that contrasts sharply with traditional batch processing methods like spin coating. The R2R method minimizes initial investment costs by eliminating the need for multiple, separate processing steps, thereby reducing equipment and maintenance expenses. Additionally, by promoting the use of low-cost, high-performance materials such as carbon inks in place of expensive precious metals such as evaporated gold, R2R directly decreases the costs associated with raw materials. The process’s scalability automation and reduction in material waste potential further amplify these savings by enabling rapid production expansion without a proportional increase in operational costs; as production scales up, the cost per unit drops.

R2R coating’s compatibility with flexible substrates broadens its applicability, facilitating integration into diverse applications beyond traditional rigid panels, fostering innovation in portable energy solutions, building-integrated photovoltaics, and aerospace^2^ broadening their use and integrating solar power into everyday environments more aesthetically and functionally^3^. By enabling continuous, high-speed production of perovskite layers, R2R technology addresses scalability directly, offering a pathway to meet growing demand for renewable energy. Rapid, large-scale production without sacrificing quality or efficiency positions R2R technique as key in widespread adoption of perovskite PV technology. The advantages of R2R coating extend to environmental benefits, minimizing waste and optimizing resource use, contributing to a more sustainable production model aligned with global environmental goals^4^. Additionally, R2R processes are more energy-efficient than batch processing methods as they continuously handle materials, avoiding the repetitive heating, cooling, and equipment start-ups of batch cycles. This efficiency not only reduces energy waste and operational inefficiencies but also further decreases the carbon footprint of solar cell production^5^.

Despite the extraordinary attributes of this technique, transition from spin coating to R2R coating in the fabrication of perovskite PV presents a range of complexities. Contrary to the controlled conditions of laboratory-based spin coating in glovebox or under fume hood, R2R coating is executed on a much larger scale in open space and requires meticulous management of coating parameters. For instance, in the slot-die R2R technique, this includes precise control over the syringe pump injection rate to achieve the required wet film thickness, drying process, appropriate shim thickness and design, and proper meniscus guide height to maintain a stable meniscus and achieve film uniformity across extended substrates. Continuous adjustments are required to accommodate variations in substrate properties, including adjustments to surface energy, as well as to environmental conditions, such as maintaining humidity below 30% RH and temperature between 20-30 °C^6–8^. Challenges intensify during drying and annealing stages, where maintaining a delicate balance of temperature, airflow, and line speed is crucial to prevent defects^8–10^.

## Entirely R2R-coated perovskite cells and modules

Transitioning from lab-scale spin-coating to fully R2R coated perovskite solar cells demands a coating technique that not only aligns with inline R2R processes but also leads to the production of efficient solar cells. Slot-die coating stands out for its versatility in both S2S and R2R processes, allowing for the optimization of each layer initially on a S2S basis before transitioning to R2R. This method is paramount for R2R production due to its exceptional process control and adaptability. It offers precise control over coating thickness and quality and leverages visco-capillary model to predict meniscus stability and film uniformity, considering the precursor solution’s characteristics such as viscosity, surface tension, and density, alongside coating parameters like shim design, meniscus height, coating speed and drying process. This precision ensures all the layers consistent quality, crucial for achieving high-efficiency cells, making R2R slot-die coating the premier choice for efficient and scalable solar cells in continuous manufacturing^11,12^. However, most research on R2R-coated perovskite solar cells uses vacuum evaporation for the top electrode, with few attempts at solution processing, which is incompatible with R2R due to time constraints, costs and high temperatures unsuitable for flexible substrates^13^.

**Fig. 1:**
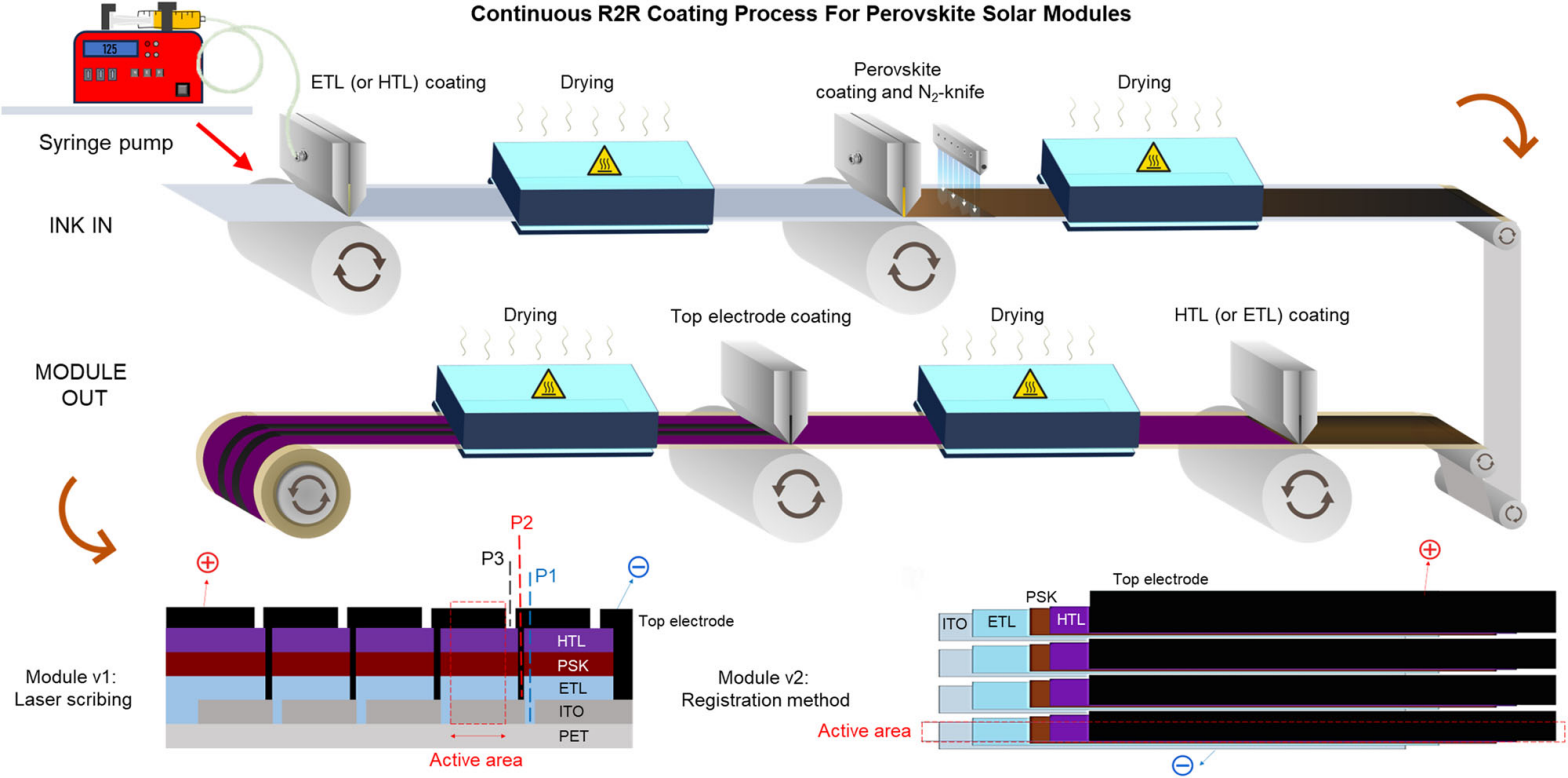
A schematic of R2R fabrication for perovskite solar modules using two fabrication approaches: laser scribing and registration. In the laser scribing method, a laser-equipped R2R coater precisely scribes the transparent conductive oxide (P1), the layer beneath the perovskite (P2), and the top electrode (P3) to provide the needed interconnections. Conversely, the registration method utilizes strip-coating, with each layer carefully aligned using a registration system to ensure accurate placement and interconnections. This approach requires a meticulous design to achieve the highest possible geometrical fill factor, presenting a greater challenge compared to laser patterning. PET Polyethylene terephthalate, ITO Indium tin oxide, N2 Nitrogen, PSK perovskite, ETL electron transporting layer, HTL hole transporting layer.

Fortunately, recent breakthroughs have successfully shifted perovskite solar cell production from partially R2R coating/ partially vacuum-processed top electrodes to a fully R2R coated process, using carbon for the top electrode, bypassing expensive metals like gold or silver, and advancing the scalability and sustainability of inline continuous solar manufacturing. Figure 1 presents a schematic of the entirely R2R coating process for perovskite solar modules. The schematic illustrates how two distinct versions of the module can be achieved through the R2R coating technique. The first version requires laser patterning to establish P1, P2, and P3 within the module. In contrast, the second version eliminates the need for laser or mechanical scribing—excluding the patterning of the ITO substrate—and instead relies on the strategic alignment of strips to fabricate the perovskite module.

Our team achieved a milestone by creating the world’s first entirely perovskite solar cells under ambient room conditions in open space, with a PCE of 10.8%^14^. This work enabled the application of all layers, including the top electrode, by utilizing carbon ink derived from inexpensive and low-toxicity solvents for slot-die R2R coating. Although carbon may not inherently surpass the electrical conductivity of traditional materials like gold, it offers a competitive alternative by striking a balance between conductivity, cost-effectiveness, and environmental sustainability. This balance is further enriched by carbon’s chemical stability, moisture resistance, and mechanical strength, which collectively enhance the durability and performance of the solar cells, positioning carbon as a viable option that supports the sustainability goals of advanced photovoltaics.

Groundbreaking work by Weerasinghe et al. advanced the field further by transitioning from single-strip cells to multi-strip modules. They employed gravure, slot-die and screen printing R2R coatings to create the first fully R2R perovskite modules, achieving a PCE of 15.5% for individual cells and 11% for serially interconnected^15^. They reported successfully transition from R2R perovskite cells to modules, using optimized fabrication parameters to create large-area modules. Employing a combination of R2R techniques—gravure for the ETL and carbon top electrode, slot-die for perovskite and HTL, and screen printing to apply transparent silver for charge-collection grids—they efficiently produced perovskite modules with integrated charge-collection capabilities.

They tackled key technical challenges in R2R coating for perovskite devices by introducing the Printing Friendly Slot-Die (PFSD) coating method under ambient air. This innovation enhanced perovskite layer quality through precursors optimizations and by employing edge-blowing techniques, ensuring rapid conversion to the desired structure suitable for R2R time constraints and achieving uniform coatings over a significant length. Edge blowing not only improved perovskite quality and device reliability but also enhanced humidity resistance, making PFSD a viable method for cost-effective production. Addressing the challenge of high-volume production, they implemented a programmable R2R slot-die coater for automated operation, enabling the daily fabrication of thousands of unique perovskite cells. Recognizing manual characterization as impractical at this scale, they introduced an automated R2R tester capable of assessing over ten thousand cells daily, with device parameters calculated and recorded online for swift analysis. This innovative approach allowed for rapid optimization of vacuum-free perovskite devices by efficiently exploring a wide range of fabrication parameters, resulting in the highest possible performance they can achieve in a shorter timeframe. Additionally, their cost model estimated production costs for perovskite solar cells to be lower than traditional methods but still higher than mass-produced silicon solar cells priced below $0.30 per watt. Nonetheless, with their estimation placing perovskite solar cells at $0.7 per watt, further reductions are necessary for the technology to achieve widespread affordability and large-scale commercialization.

While recognizing their commendable accomplishments, particularly in the development of low-cost, efficient, fully R2R-coated perovskite modules, there is an opportunity to further explore an aspect critical to scaling up perovskite devices: environmental considerations. While they employed non-toxic deionized DI water for the ETL, the solvents used in other parts of the process are highly toxic, with some having strict exposure limits in open spaces. Specifically, dimethylformamide DMF was used for the perovskite layer, and chlorobenzene and dichlorobenzene were used for the HTLs. This raises important safety and environmental issues that need to be addressed to ensure the sustainable development and commercial viability of perovskite solar cell technology.

## Future of R2R perovskite devices

The capability of R2R to efficiently produce high-quality, uniform films over large areas positions it as the preferred method for future advancements in the field. Envisioning the future of R2R coating for perovskite solar cells, the quest for scalable, efficient, and environmentally sustainable solar energy production takes center stage. Enhancements in performance are attainable by refining R2R coating parameters to overcome technical limitations and optimizing module design and interconnections to get the highest possible geometrical fill factor. Such innovations aim to maximize the active area of the solar cells, optimizing the performance of the devices. Moreover, the integration of quality control mechanisms within the R2R process poses its own set of challenges. Inline monitoring systems must be developed to provide real-time feedback on the quality of the perovskite layers, enabling immediate adjustments to the fabrication parameters. This requirement not only increases the complexity of the R2R setup but also necessitates advanced sensor technologies capable of detecting a wide range of material properties under dynamic processing conditions.

Furthermore, addressing the environmental impact of perovskite PV production remains a significant challenge. Future advancements in R2R technology must directly tackle toxicity concerns by integrating non-toxic solvents across all device layers and exploring substitutes for lead within the perovskite material itself. This pursuit of greener alternatives is essential not only for regulatory and environmental reasons but also to mitigate health risks, ensure safer production environments, and improve solar panel recyclability^16–18^.

Shifting towards non-toxic materials and processes is crucial to align the rapid production capabilities of R2R coating with sustainability goals. The sustainability challenges for large-scale R2R coating of perovskite solar cells extend beyond this shift to include minimizing material waste, reducing the energy consumption of the coating process, ensuring the durability of the solar cells for long-term use, and developing effective recycling and disposal methods for environmental safety and resource recovery. Establishing efficient recycling protocols is imperative for managing waste and recovering valuable materials, reinforcing the industry’s commitment to sustainability. These protocols might involve mechanical and chemical processes tailored to efficiently separate and recover perovskite and other valuable materials from spent modules. Although recent studies provide a framework for recycling strategies that minimize environmental impact while maximizing material recovery, particularly on glass substrates^19^, the practical application of such protocols to R2R coated perovskite solar cells on flexible substrates at a large scale remains a significant challenge. This highlights a crucial gap in the current sustainability efforts as the perovskite photovoltaic sector scales up. Ultimately, the future of perovskite solar modules hinges on a balance between technological innovation, environmental responsibility, and economic viability^20^. Additionally, the transition to R2R fabrication necessitates a reassessment of substrate materials. While flexible substrates offer the advantage of enabling new applications, they also introduce variability in surface properties, temperature durability and mechanical stability, which can affect coating uniformity, and device performance. Developing substrates that are both compatible with R2R processing and conducive to high-efficiency perovskite device fabrication requires significant material innovation and optimization. Specifically, the selection of substrate materials in R2R printing plays an essential role in environmental sustainability. Advanced materials such as recyclable or biodegradable substrates can substantially reduce the environmental footprint. Advanced materials, like Polylactic Acid (PLA), recycled PET (r-PET), cellulose-based materials, and biomimetic substrates, can substantially reduce the environmental footprint. Innovations in substrate technology, as highlighted by Recent research, show the potential for using these materials to not only support the integrity and performance of perovskite solar cells but also align with environmental objectives^21,22^. The integration of environmental considerations into the selection of substrates and the formulation of recycling protocols is essential to advance the R2R fabrication of perovskite solar cells towards a more sustainable future.

In conclusion, R2R fabrication offers a promising pathway for scaling up perovskite PV cell and module production, focusing on continuous coating, cost reduction, and performance enhancement. However, transitioning from lab-scale spin coating to industrial-scale R2R processes presents technical challenges. Innovations in materials, R2R processes, and module design are crucial for improving efficiency, stability, and environmental sustainability. Collaborations between academia and industry, along with the establishment of global standards are essential for transitioning perovskite technology to commercial scale. By embracing these strategies, R2R coating can significantly transform the renewable energy landscape, facilitating progress towards a sustainable and energy-efficient future.

## References

[1] Dou B (2018). Roll-to-roll printing of perovskite solar cells. ACS Energy Lett..

[2] Tian, R., Zhou, S., Meng, Y., Liu, C. & Ge, Z. Material and device design of flexible perovskite solar cells for next‐generation power supplies. *Adv. Mater*., 2311473 (2024). 10.1002/adma.202311473 38224961

[3] Benitez-Rodriguez JF, Chen D, Gao M, Caruso RA (2021). Roll‐to‐roll processes for the fabrication of perovskite solar cells under ambient conditions. Sol. RRL.

[4] Urbina A (2020). The balance between efficiency, stability and environmental impacts in perovskite solar cells: a review. J. Phys.: Energy.

[5] Schileo G, Grancini G (2021). Lead or no lead? Availability, toxicity, sustainability and environmental impact of lead-free perovskite solar cells. J. Mater. Chem. C..

[6] Li H (2022). Fully roll-to-roll processed efficient perovskite solar cells via precise control on the morphology of PbI2: CsI layer. Nano-Micro Lett..

[7] Zuo C, Vak D, Angmo D, Ding L, Gao M (2018). One-step roll-to-roll air processed high efficiency perovskite solar cells. Nano Energy.

[8] Burkitt D (2020). Roll-to-roll slot-die coated P–I–N perovskite solar cells using acetonitrile based single step perovskite solvent system. Sustain. Energy Fuels.

[9] Ham DS (2021). Influence of drying conditions on device performances of antisolvent-assisted roll-to-roll slot die-coated perovskite solar cells. ACS Appl. Energy Mater..

[10] Liu WW, Wu TH, Liu MC, Niu WJ, Chueh YL (2019). Recent challenges in perovskite solar cells toward enhanced stability, less toxicity, and large‐area mass production. Adv. Mater. Interfaces.

[11] Rolston N (2020). Rapid open-air fabrication of perovskite solar modules. Joule.

[12] Rolston N (2021). Perspectives of Open-air processing to enable perovskite solar cell manufacturing. Front. Energy Res..

[13] McGovern L, Garnett EC, Veenstra S, van der Zwaan B (2023). A techno-economic perspective on rigid and flexible perovskite solar modules. Sustain. Energy Fuels.

[14] Beynon D (2023). All‐Printed Roll‐to‐Roll Perovskite Photovoltaics Enabled by Solution‐Processed Carbon Electrode. Adv. Mater..

[15] Weerasinghe HC (2024). The first demonstration of entirely roll-to-roll fabricated perovskite solar cell modules under ambient room conditions. Nat. Commun..

[16] Vidal R (2021). Assessing health and environmental impacts of solvents for producing perovskite solar cells. Nat. Sustaina..

[17] Miao Y (2023). Green solvent enabled scalable processing of perovskite solar cells with high efficiency. Nat. Sustain..

[18] Zhang H (2023). Lead immobilization for environmentally sustainable perovskite solar cells. Nature.

[19] Tian X, Stranks SD, You F (2021). Life cycle assessment of recycling strategies for perovskite photovoltaic modules. Nat. Sustain..

[20] Zhu P (2024). Toward the commercialization of perovskite solar modules. Adv. Mater..

[21] Lu Z (2020). Highly flexible and transparent polylactic acid composite electrode for perovskite solar cells. Sol. RRL.

[22] Gao L (2019). Flexible, transparent nanocellulose paper-based perovskite solar cells. npj Flex. Electron..

